# Asymmetric Effect of Mechanical Stress on the Forward and Reverse Reaction Catalyzed by an Enzyme

**DOI:** 10.1371/journal.pone.0101442

**Published:** 2014-07-07

**Authors:** Collin Joseph, Chiao-Yu Tseng, Giovanni Zocchi, Tsvi Tlusty

**Affiliations:** 1 Department of Physics and Astronomy, University of California Los Angeles, Los Angeles, California, United States of America; 2 Institute for Advanced Study, Princeton, New Jersey, United States of America; University of Zurich, Switzerland

## Abstract

The concept of modulating enzymatic activity by exerting a mechanical stress on the enzyme has been established in previous work. Mechanical perturbation is also a tool for probing conformational motion accompanying the enzymatic cycle. Here we report measurements of the forward and reverse kinetics of the enzyme Guanylate Kinase from yeast (*Saccharomyces cerevisiae*). The enzyme is held in a state of stress using the DNA spring method. The observation that mechanical stress has different effects on the forward and reverse reaction kinetics suggests that forward and reverse reactions follow different paths, on average, in the enzyme's conformational space. Comparing the kinetics of the stressed and unstressed enzyme we also show that the maximum speed of the enzyme is comparable to the predictions of the relaxation model of enzyme action, where we use the independently determined dissipation coefficient 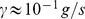 for the enzyme's conformational motion. The present experiments provide a mean to explore enzyme kinetics beyond the static energy landscape picture of transition state theory.

## Introduction

The catalytic cycle of enzymes is often coupled to a mechanical cycle of conformational changes, or deformations, of the enzyme. This property reflects the generality of the induced fit mechanism envisioned by Koshland fifty years ago [1]. Enzymes generally deform when binding the substrates and releasing the products of the reaction they catalyze. On the other hand, the canonical description of the kinetics of enzymatic reactions, Michaelis-Menten (MM) kinetics [2], does not explicitly refer to conformational motion. Indeed, MM kinetics is often linked to an effective, static energy landscape of the transition state theory. In its simplest form, MM kinetics follows from the reaction scheme: 
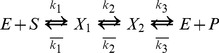
(1)where 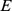: enzyme; 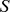: substrate; 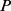: product; 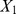: enzyme-substrate complex; 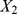: enzyme-product complex. Under quasi-equilibrium assumptions [3] the catalytic kinetics at 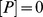 is:
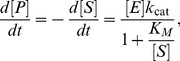
(2)where 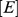 is the total enzyme concentration, 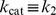 the catalytic rate, and 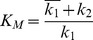 the MM constant. A similar expression applies for the backward reaction, where the roles of substrate and product are exchanged.

**Figure 1:**
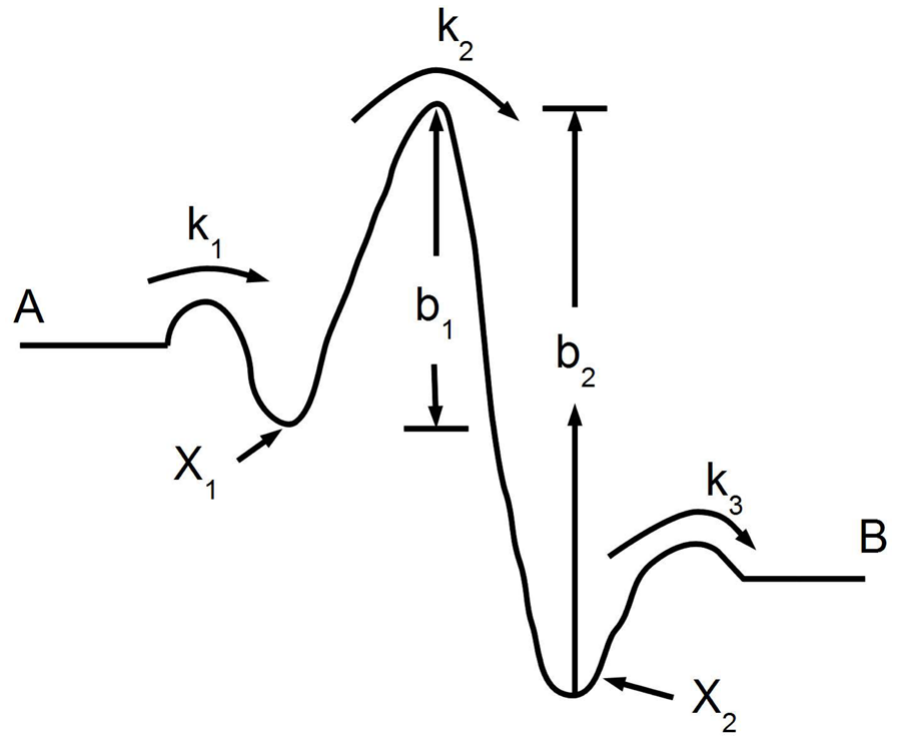
The canonical energy landscape for an enzymatic reaction. 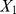
 is the bound state representing the enzyme - substrate complex; 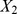 represents the enzyme - product complex.

The corresponding energy landscape Fig. 1 links the rates in (1) with the energy barriers, e.g. 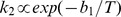 etc. Experimentally, one studies the kinetics of the forward or the reverse reaction by varying the concentration (the chemical potential) of substrates and products, which corresponds to shifting the energy levels A and B in Fig. 1, while the rest of the energy landscape (i.e. 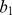 and 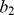) remains the same. Implicit in the one-dimensional description summarized by (1) and Fig. 1 is that forward and reverse reactions follow the same path through the energy landscape, in opposite directions. This assumption imposes certain restrictions on the effect that a perturbation of the enzyme can have on the forward and reverse kinetics.

**Figure 2:**
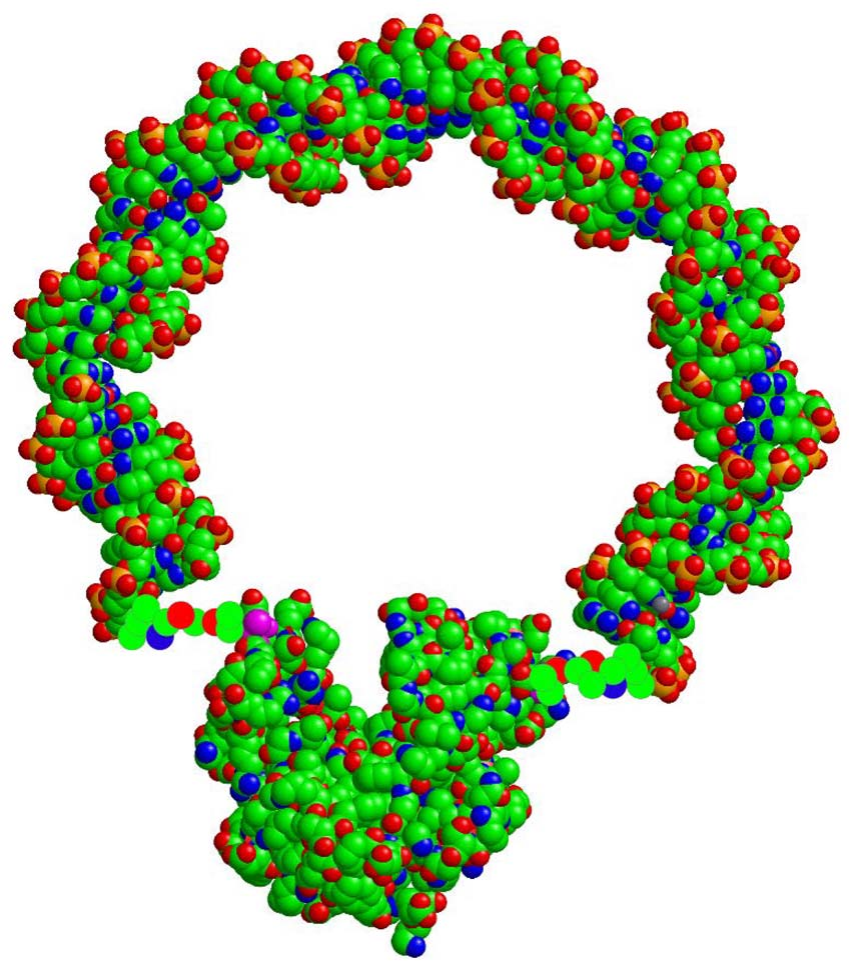
Cartoon of the yeast GK-DNA chimera (in the “open" state) with the DNA spring attached at the sites 60 and 139. Upon binding of the substrate GMP, the two lobes of the enzyme close on each other. This 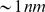 size conformational motion is a classic example of induced fit. The enzyme structure is PDB 1EX6; the DNA is from the MD simulations of Lankas et al [38], and is kinked in the middle.

In this work, we compare the kinetics for the forward and reverse reactions catalyzed by the enzyme Guanylate Kinase (GK) under different states of mechanical stress. The role of mechanical stress in protein dynamics has been considered early on, originally in the context of cooperative allosteric transitions [4], [5]. Today, mechano-sensitive enzymes is a topic of much research interest [6]–[8]. Here, we ask a simple question about the enzyme under mechanical stress: given a measured effect of the stress on the forward reaction, what is the effect of the same mechanical stress on the reverse reaction? This question could not be posed experimentally before, because until recently there were no practical methods available to apply a non-destructive mechanical stress on an enzyme while simultaneously measuring the kinetics of the catalyzed reaction. Presently such measurements can be obtained with the enzyme-DNA chimeras (Fig. 2), where a DNA molecular spring attached to the enzyme exerts a force in a known direction [9]–[11].

## Results

In the experiments we compare two hybridization states of the same chimera sample: the chimera hybridized to two separate 30mer or 28mer strands (producing a DNA spring with a nick or a gap in the center) and hybridized to one 60mer strand (no nick in the DNA spring). The first (nicked or gapped) state is a low to zero stress state [12], [13], the second (non-nicked) state is higher stress. Otherwise these two states are chemically identical: same amount of DNA in proximity to the enzyme, same charge, very similar conformation of the DNA spring, etc. Therefore, if “non-mechanical" effects [13], [14] of the DNA spring on the enzyme are present (steric, electrostatic, etc.), they must be essentially identical for the two states (nicked and non-nicked). This point is further discussed later.

**Figure 3:**
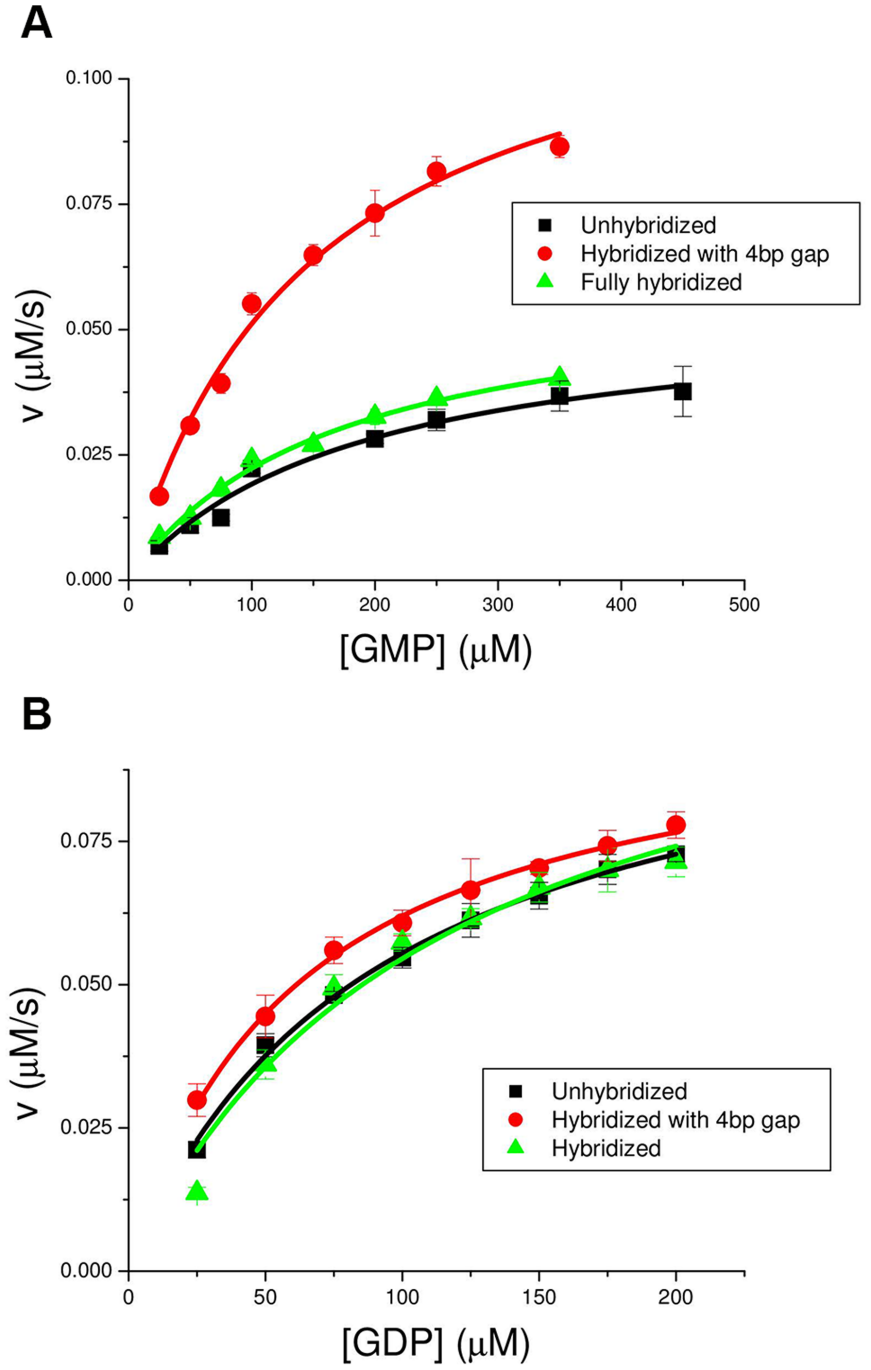
“Low Mg” titration curves (initial reaction speed vs. initial substrate concentration) for the yeast GK chimera in the absence (circles) and presence (triangles) of mechanical stress. Circles: double-stranded DNA spring with a 4 bp central gap; triangles: double-stranded DNA spring with no gap or nick. Squares: ssDNA spring; this is a confusing control explained in the text. Titrations were performed under the same “low Mg" buffer conditions (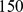 mM KCl and 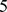 mM MgCl_2_) and with the same chimera sample. Here and for Fig. 4, each experimental point represents the average of 4–5 measurements (with the same chimera sample); error bars are the standard deviation. Similar titration curves were also obtained with 3 different (independent constructions of) chimera samples (data not shown), with the same result within experimental error. a) GMP titration for the forward reaction. Enzyme concentration was 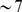 nM and initial ATP concentration was 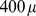
M. b) GDP titration for the reverse reaction. Enzyme concentration was 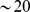 nM (3 times larger than in (a)) and initial ADP concentration was 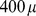
M.

With this paradigm, Fig. 3 shows that the mechanical stress slows down the forward reaction (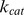 is reduced by a factor 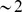), while the same stress has no effect on the reverse reaction. In more detail, Fig. 3a shows the result of GMP titration experiments for the forward reaction: 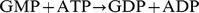. The initial speed of the reaction (measured through a coupled enzymatic assay, see Methods) is plotted against the initial GMP concentration. Conditions were: enzyme concentration 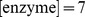 nM, initial ATP concentration 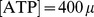
M, zero products conc. initially, 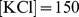 mM, 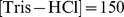 mM, pH 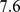, and 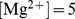 mM. The circles show the reference zero stress state (there is a 4 bases long ss gap in the middle of the ds DNA spring); the triangles show the stressed state (ds DNA spring with no gaps). The squares show the measurements with the DNA spring in the ss form. They confuse rather than clarify the issue at hand but we report them for completeness. Evidently, the DNA spring in the ss form partially inhibits the enzyme, the inhibition being lifted by hybridization of the DNA, in the absence of stress (i.e. with the gap). Our interpretation of this phenomenon, which we have documented before with Guanylate Kinase from TB [13], [14], is that the ss DNA spring interacts with the nucleotide binding site of the enzyme, leading to partial inhibition. Hybridization removes the DNA from the surface of the enzyme, lifting the inhibition. Such “non-mechanical" effects are absent in the case of an enzyme which is not nucleotide-binding [10]. We will come back later to the peculiarity that the inhibitory effects of the mechanical stress (triangles) and of the ss DNA (squares) are identical: this cannot be a coincidence.

Fig. 3b shows the result of GDP titration experiments for the reverse reaction: 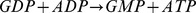. Conditions were: enzyme concentration 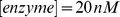 (3 times the concentration used for the forward reaction), initial ADP concentration 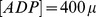
M, same salt conditions as Fig. 3a. There is no effect of the mechanical stress on the reverse reaction. Overall, the reverse reaction is a factor 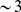 times slower (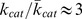), consistent with previous measurements by Li et al [15]. The lines in Fig. 3 are fits using the two-substrate MM expression: 
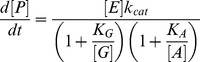
(3)where 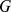 and 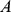 stand for GMP and ATP (or GDP and ADP for the reverse reaction), and we neglect the interaction term between the two substrates for simplicity. Combining GMP and ATP titration curves (the latter reported in Methods) these fits determine the three parameters 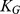, 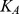, 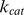, which are reported in Tables 1 and 2. In the absence of stress, this set of kinetic parameters has been determined previously, and probably more accurately than we do here, for the wild type yeast GK by Li et al [15], who also studied the effect of different Mg^2+^ concentrations; our objective here is to examine the effect of mechanical stress.

**Table 1:** The kinetic parameters 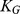, 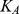, and 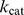 extracted from the titration data for the forward direction (formation of GDP and ADP) and the reverse reaction (formation of GMP and ATP) in high Mg conditions.

	Hybridization state	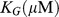	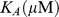	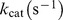
Forward Reaction	unhybridized	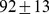	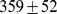	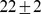
	hybridized with 4 bp gap	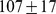	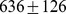	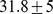
	fully hybridized	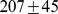	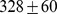	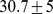
Reverse Reaction	unhybridized	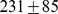	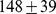	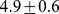
	hybridized with 4 bp gap	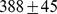		
	fully hybridized	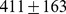		

High Mg buffer conditions are 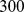 mM KCl, 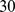 mM MgCl_2_, 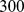 mM Tris-HCl at pH 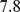.

**Table 2:** The kinetic parameters 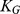, 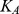, and 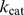 extracted from the titration data for the forward and reverse reactions in low Mg conditions.

	Hybridization state	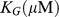	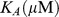	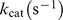
Forward Reaction	unhybridized	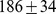	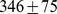	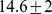
	hybridized with 4 bp gap	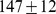	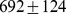	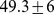
	fully hybridized	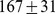	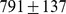	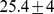
Reverse Reaction	unhybridized	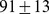	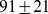	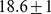
	hybridized with 4 bp gap	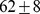	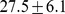	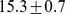
	fully hybridized	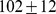	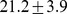	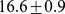

Low Mg buffer conditions are 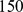 mM KCl, 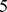 mM MgCl_2_, 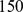 mM Tris-HCl at pH 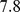.

**Figure 4:**
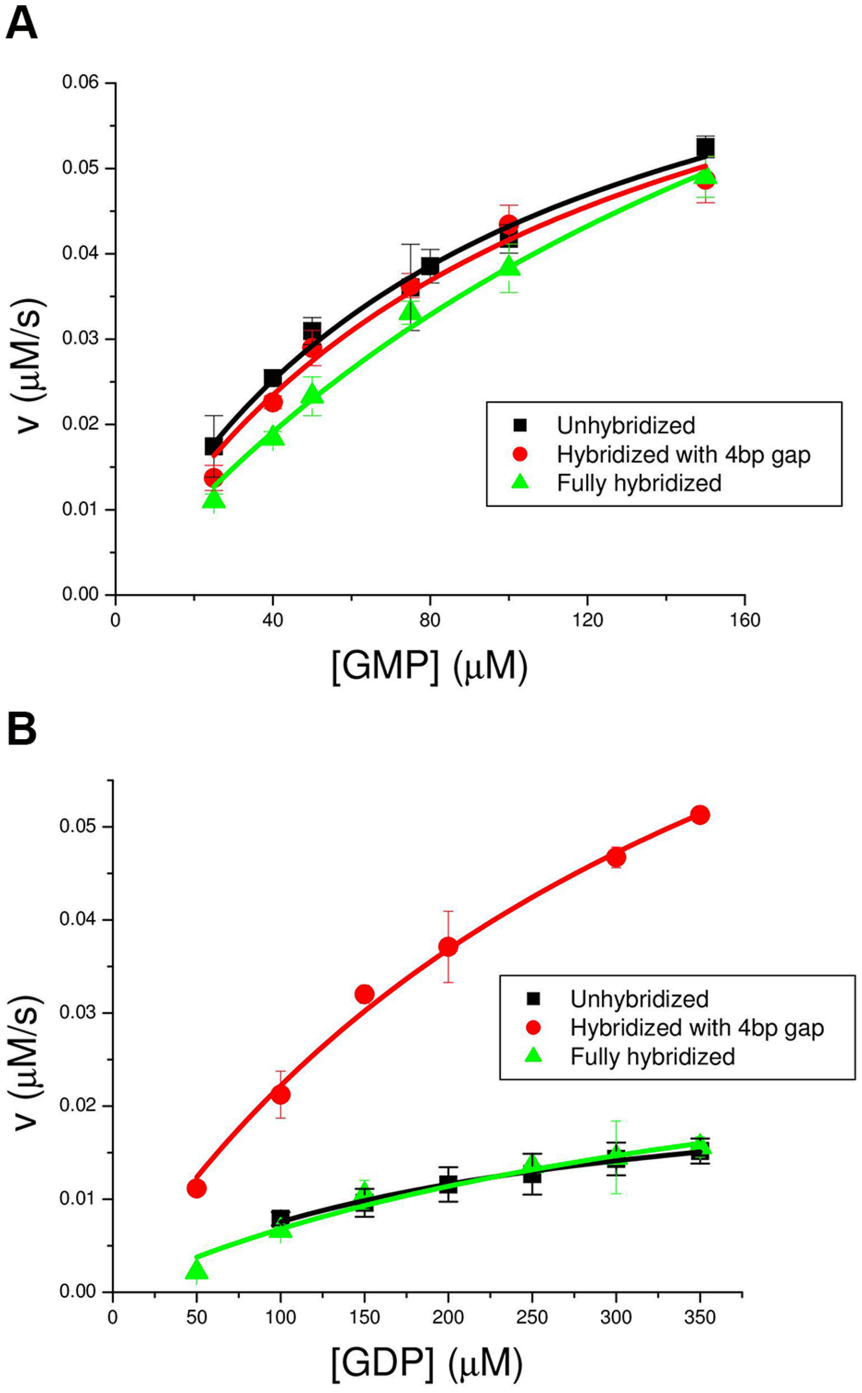
“High Mg” titration curves (initial reaction speed vs. initial substrate concentration) for the yeast GK chimera in the absence (circles) and presence (triangles) of mechanical stress. Circles: double-stranded DNA spring with a 4 bp central gap; triangles: double-stranded DNA spring with no gap or nick. Squares: ssDNA spring. Titrations were performed under "high salt" buffer conditions: 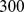 mM KCl and 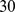 mM MgCl_2_. a) GMP titration curves for the forward reaction. Conditions were the same as in Fig. 3: enzyme concentration was 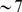 nM and and initial ATP concentration was 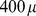
M. b) GDP titration curves for the reverse reaction. Enzyme concentration was 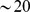 nM and initial ADP concentration was 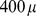
M.

To summarize: mechanical stress in the direction defined by the DNA spring attachments of Fig. 2 inhibits the forward reaction but not the reverse reaction, under conditions were 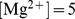 mM (“low Mg"). We repeated the measurements for "high Mg" conditions (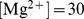 mM), and found the opposite behavior (Fig. 4): now the same mechanical stress inhibits the reverse reaction, but has no effect on the forward reaction. The behavior for “low Mg" and “high Mg" is remarkably symmetric (Figs. 3, 4). Now we explain what we think is interesting in the above phenomenology.

First let us note that the effect of the DNA spring can in no way be characterized as biasing an active/inactive (e.g. folded/unfolded) equilibrium of the enzyme. Previous experiments with the DNA spring show that in general different parameters of the enzymatic cycle are affected differently by the mechanical stress [10], [13]. The present measurements show that the forward and backward reaction are affected differently by the same DNA spring. In fact, all our results so far show that there is merit in considering the enzyme as essentially continously deformable.

In the context of the 1-D energy landscape of Fig. 1, a perturbation of the enzyme (such as our mechanical stress) can change the forward rate 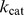 (i.e. the barrier height 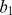) in one of two ways: by changing the transition state level, or by changing the level 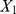, relative to the rest of the landscape. In the first case, the reverse rate 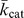 necessarily also changes, while in the second case it does not. Thus the measurements of Fig. 3 would imply that the effect of mechanical stress is to lower the level 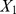 relative to the rest, thus increasing 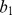. However, the level 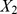 must be unaffected since 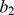 does not change (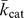 does not change). On the contrary, the measurements of Fig. 4 would imply that for “high Mg", the effect of the same stress is to lower the level 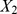 while the level 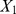 is unaffected. Both conclusions seem unreasonable: if mechanical stress shifts the level 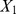, it must be that the stress distorts the nucleotide binding pocket; then both levels 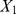 and 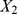 should shift (they represent the same binding sites). In our view, a more plausible representation is to abandon the one dimensional scheme (1) and allow the forward and reverse reactions to proceed, on average, along different paths.

**Figure 5:**
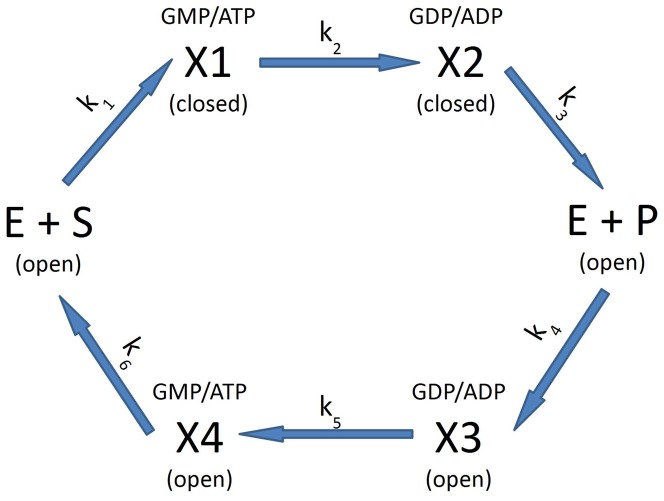
A proposed two-dimensional reaction scheme which can explain the observed effects of mechanical stress. E, S, and P stand for enzyme, substrates, and products; the upper branch refers to the forward reaction, the lower branch to the reverse reaction. Open and closed refer to the state of the enzyme; 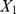 is a complex of the enzyme with GMP and ATP, and similarly for the other states.

As an example, consider the scheme of Fig. 5, where we have tentatively used the extra variable open/closed (referring to the conformational state of the enzyme) to distinguish 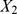 from 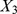 and 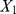 from 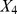. However, we are not introducing additional kinetic parameters compared to (1): there are still 6 different rates. As in (1), these rates cannot all be varied independently by perturbing the enzyme, because the equilibrium constant of the reaction dictates the value of the ratio 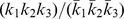 (or 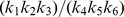 in the scheme of Fig. 5). However, it now seems possible that a mechanical stress may affect 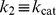 but not 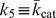, because the state 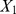 is different from the state 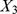. In structural terms, if we adopt the open/closed assignments of Fig. 5, we might say the mechanical stress affects the closed state but not the open state. In order to accomodate the “high Mg" data (Fig. 4) within the same scheme, we have to assume that at high Mg all the arrows in Fig. 5 are reversed, i.e. the forward reaction happens in the open state and the reverse reaction in the closed state. This is obviously an ad hoc assumption, but consider that given the remarkable symmetry displayed by the measurements in Figs. 3, 4 there must be a simple symmetry operation connecting the reaction schemes for low and high Mg. In structural terms, what is different at low and high Mg is the coordination state of the nucleotides: in solution, the dissociation constants for MgATP and MgADP are 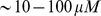, while for MgAMP and MgGMP 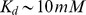 [16], [17]. The dissociation constant for MgGDP is not completely clear but probably in the neighborhood of 2 mM [18]. Under our experimental conditions, the coordination state and charge of the substrates and products (at least in solution) are therefore presumably as in Table 3, where we have chosen to list GDP as not complexed with Mg at low Mg.

**Table 3:** The coordination states for the reaction substrates and products in both low and high Mg^2+^ conditions.

Low 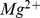		High 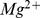	
MgATP^2−^	MgADP^−^	MgATP^2−^	MgADP^−^
GMP^2−^	GDP^3−^	MgGMP	MgGDP^−^

Since 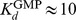 mM, in high Mg conditions, GMP is complexed with Magnesium. Whether GDP is complexed with Magnesium or not for low Mg conditions is not completely clear.

This difference must ultimately be responsible for the phenomenology displayed in Figs. 3, 4; in the context of the heuristic scheme Fig. 5 we have to assume that this difference controls the direction of the arrows in the figure.

The conjecture of Fig. 5 also rationalizes, to some extent, the coincidence of squares and triangles (unhybridized and fully hybridized chimeras) in Figs. 3, 4. Namely, ss DNA fits in the deep groove between the two lobes of the molecule (see Fig. 2); as it interacts with the nucleotide binding site, it would tend to keep the structure in the open state. Removing the DNA from the groove, by hybridization, allows the structure to access the closed state.

Let us now take a more dynamic point of view, and see whether these experimental results are compatible and indeed support the viscoelastic model of enzyme dynamics which we recently proposed [19]. This model is in the spirit of the relaxation model proposed earlier by Blumenfeld [20], and addresses explicitly the conformational motion of the enzyme during the catalytic cycle. In the case that large conformational motion takes place (forward reaction in Fig. 5, where the enzyme undergoes the mechanical cycle 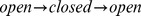), we hypothesized in [19] an effectively one-dimensional viscous kinetics, in which the open-to-closed conformational change of GK is driven by substrate binding through a force 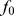, resisted by a restoring force 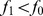. The ensemble averaged trajectory is characterized by a velocity 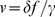, where 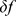 is the resultant force and 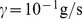 is a dissipation coefficient which we have measured for this enzyme through independent nano - rheology experiments [21].

Using notation consistent with [19], we write: 
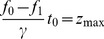
(4)for the forward (open to closed) part of the enzyme's cycle. 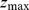 is the size of the conformational change, and 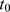 is the duration of the motion. We clarify that in this part of the paper, forward and backward refer to the open to closed and closed to open parts of the mechanical cycle of the enzyme, *not* the forward and reverse reactions. Similarly,
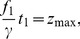
(5)where 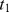 is the duration for the backward (closed to open) part of the enzyme's cycle. The cycle duration is therefore:



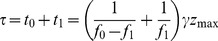
(6)Note that varying 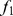 has opposite effects on 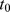 and 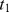, and therefore there is an extremal value 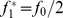 which minimizes 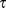. The experimental manner in which we tweak 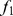 is by the DNA spring, which modifies the driving force as 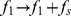, where 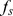 is the force exerted by the spring. This we have also measured in independent experiments [22], [23], and 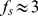 pN in our case. Then we find the ratio of cycle duration in the absence and presence of the spring: 
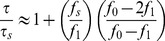
(7)where we have only kept terms first order in 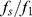.

For the reverse reaction we have a different driving force 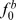 since the substrate is now different ("b" stands for backward), but the same restoring force 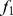, dissipation 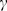, and amplitude 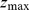. For the reverse reaction, the scheme of Fig. 5 says, in the present language, 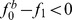, i.e. the driving force of substrate binding is not sufficient to drive the open to close transition. For the forward reaction however: 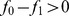 since the enzyme goes to the closed state. Further, 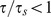 (under stress the overall rate slows down) which means (using (7)) 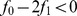. Putting everything together: 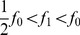
(8)


The measurements of Fig. 3a give, in this language: 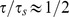; writing 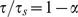, we have from (7): 
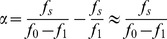
(9)(since 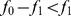 we keep only the first of the two terms). Using 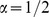 (from the measurements) we finally have:



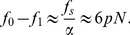
(10)We can now estimate the speed of the enzyme using (6): 
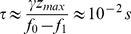
(11)using 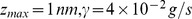 (the value reported in [21]). This value compares well with 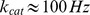 (our value for the forward reaction in the absence of stress is 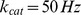, see Table 1; the value of Li et al [15] for the wild type is 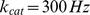).

We have the remarkable result that the relaxation model [19] predicts the speed of the enzyme based on mechanical quantities: the forces 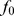 and 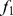 inferred from the experiments with the DNA spring and the dissipation 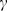 measured in an independent “nano-rheology” experiment [21].

## Discussion

In this paper we develop the idea of comparing the speed of the forward and backward reactions catalyzed by an enzyme in the presence and absence of mechanical stress, and present the first such measurements. Of the several different perturbations one could apply to the enzyme, mechanical stress seems particularly interesting since it couples directly to conformational motion. Our results are not naturally accomodated by the canonical one-dimensional reaction scheme expressed by (1) and Fig. 1. Assuming that the forward and reverse reactions follow diferent paths seems a more plausible representation of the measurements; an example of the simplest such scheme is given in Fig. 5. In the language of Fig. 5, the central point we want to make is that 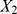 is different from 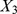, and 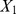 is different from 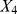; the specific assignments of Fig. 5 are completely conjectural and may or may not be true. Within the scheme of Fig. 5, we can rationalize the rather surprising "symmetric" behavior of the system for low and high Mg by adding yet another conjecture, namely that at high Mg all the arrows in Fig. 5 are reversed.

**Figure 6:**
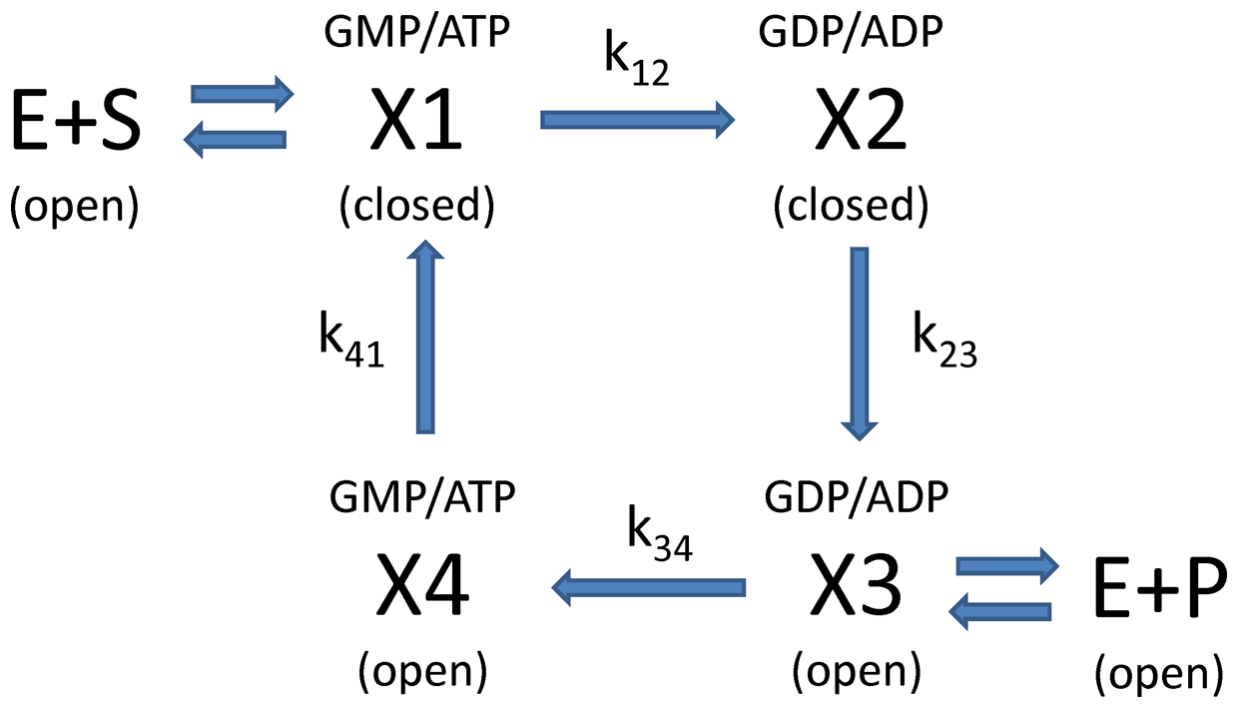
An alternative two-dimensional reaction scheme which is also consistent with the observations. Here two forms of the enzyme, open and closed, are stabilized by the presence of GDP/ADP and GMP/ATP, respectively. The essentially uni-directional arrows 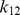 and 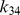 can be accomodated within standard transition state theory if one assumes, in both cases, a high barrier for the reverse process. As with the scheme of Fig. 5, we have to assume that under high Mg conditions the arrows in the loop are reversed.

On the other hand, other "two-dimensional" kinetic schemes are possible and consistent with the data; one further example is summarized in Fig. 6, and was suggested by one Referee of this paper. Fig. 6 represents a situation where there are two forms of the enzyme, termed open and closed. In the closed form, the barrier for the reverse reaction (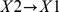) is very high, making the step 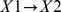 essentially irreversible. Similarly, in the open state the barrier for the forward reaction (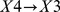) is very high. Further, the presence of the substrates (GMP/ATP) stabilizes the closed form, while the presence of the products (GDP/ADP) stabilizes the open form. Similarly to the scheme of Fig. 5, we then have to assume that mechanical stress affects the barrier in the closed state but not in the open state. Further, we must assume that under high Mg conditions the arrows in the loop of Fig. 6 are reversed.

Let us now pause to clarify one basic point and reflect on another. Some may dislike the uni-directional arrows in Figs. 5, 6. However, they can easily be reconciled with standard transition state theory by a judicious choice of barriers in a sufficiently high - dimensional (here, higher than one dimension) energy landscape. But more generally, there is no contradiction with microscopic time reversal symmetry, much less with detailed balance. The rates in (1) etc. are ensemble averaged quantities, and they imply an underlying dissipation: for instance, in the Kramers theory which connects 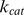 to the energy landscape Fig. 1 this rate is inversely proportional to an effective viscosity [24]. Compared to (1), the scheme of Fig. 5 essentially adds one more coordinate to the problem, here identified, somewhat arbitrarily, with the open/closed conformational states. Within the energy landscape context, adding one or more dimensions to the single "reaction coordinate" of Fig. 1 is not something new, see for example the recent work [25] in the context of mechanical unfolding. However, and on a more general level, in the case where large conformational motion accompanies enzyme action, a description in terms of a static energy profile seems unsatisfactory to us. The whole notion of an energy landscape is rather vague in this case. The "reaction coordinate" of Fig. 1 should really be time (i.e. the figure pictures the free energy of a sequence of events), but the same coordinate is also taken to mean space for the purpose of calculating rates with the Kramers theory (one takes gradients of the free energy in Fig. 1 and so on). Pehaps if the enzyme deforms at constant speed along the reaction coordiante (the relaxation model) the two views can be reconciled, but in any case these difficulties motivate us to attempt to incorporate explicitly the enzyme's motion into the description of the kinetics. Here we do this through the viscoelastic relaxation model [19], itself an explicit realization of Blumenfeld's relaxation model [20]. From the kinetic measurements with and without stress we obtain estimates for the driving forces of the mechanical cycle of the enzyme, and from these and the independently measured dissipation coefficient 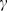 we construct the maximum rate of the enzyme 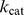, which is roughly correct. A noteworthy fact is that the order of magnitude of the dissipation coefficient 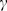 is the same for different experiments and enzymes, as results from our measurements on GK [21], the earlier AFM indentation experiments of the Hansma group [26], and measurements on the gating motion of a 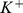 channel [27]. The force-velocity curve of the motor kinesin, when interpreted in terms of internal friction, as suggested by Howard [28], gives also a similar value 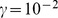
g/s (Fig. 2 of [28]).

Other frameworks have been proposed to deal with force induced deformation, primarily in the context of receptor - ligand unbinding and more specifically "catch - bonds" [29]. One approach is to increase the effective dimensionality of the energy landscape, thus introducing different possible pathways for the process in question [30]–[33]. This is similar to Fig. 5. Another approach is through the concept of force - induced allostery [34], [35], though the two are related at the model level [36].

On a more technical note, for the measurements we always compare different hybridization states of the same chimera preparation, so that the enzyme concentration in the samples is the same by construction. Otherwise it is difficult to ensure exactly equal concentrations of enzyme. Further, we take as the reference low stress state the nicked chimera or equivalently the chimera with a 4 base long ss gap in the DNA spring: this is necessarily a zero stress state and the results are identical for this and the nicked case. On the other hand, we cannot take as reference zero stress state the unhybridized (ss) chimera, because partially hybridizing the ss chimera, in the absence of mechanical stress (i.e. leaving a ss gap), also has an effect on the activity. We have documented these "non-mechanical" effects before [13], [14]; it is not surprising that different conformations of the DNA spring (a flexible coil in the ss form, a rigid rod with flexible joint in the middle for the gapped case) may interact differently with the enzyme and the substrates. For example, the negatively charged chimera DNA may pose a barrier to the diffusion of the negatively charged substrates and products in and out of the binding site. Or perhaps the ss DNA keeps the enzyme in the open state by blocking the open to closed conformational motion. We have recently characterized a different enzyme-DNA chimera where the enzyme is not a nucleotide binding protein, and find no such "non-mechanical" effects [10].

In summary, we believe that the nicked chimera (or equivalently the gapped chimera) is the correct low stress reference state to compare to. The conformation of the nicked and non-nicked DNA spring are so similar (both semi-flexible rods with the same charge) that any non-mechanical effect must be the same in both cases. Therefore we compare triangles and circles in Figs. 2, 3 to extract the effect of mechanical stress on the enzyme. The experimental result is that the forward and reverse reactions are affected differently by the mechanical stress. A plausible explanation is that the forward and reverse reactions follow different paths, not the same path in reverse. Further, we argue that the maximum speed of the enzyme, 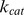, is either limited or close to being limited by the enzyme's relaxation dynamics.

This enzyme (GK) was chosen due to our previous experience with it, and because the forward and backward reactions are easily quantified. In the future, we hope to extend similar measurements to other enzymes, probing the relation of conformational motion to enzyme kinetics.

## Materials and Methods

In these experiments we use an enzyme-DNA chimera synthesized using Guanylate Kinase from *Saccharomyces cerevisiae* (gene GUK1) [13], [14]. This enzyme is structurally almost identical to GK from *Mycobacterium tuberculosis* (TBGK) with which our previous work was performed. For both enzymes, substrate binding is accompanied by large (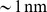) conformational motion from the “open" to the “closed" conformation (Fig. 2). However, yeast GK is about 10 times faster than TBGK [15]. We use site-directed mutagenesis to introduce two Cys residues on the surface of the enzyme at positions 60 and 139 of the chain to echo the 75/171 position of [13], where the DNA spring works directly against the open to closed conformational change of the enzyme (see Fig. 2 for an illustration). The modified gene, which also contains a His-tag to facilitate purification of the protein, is then expressed in E. coli and the enzyme purified on Ni-NTA beads. To construct the chimera, we use two 30 bases long DNA strands with amino terminal modifications at the 5′ and 3′ end respectively. The amino-functionalized DNA strand is attached to the Cys residues through a hetero-bifunctional crosslinker (NHS-PEO2-Maleimide), which reacts with the amine group on the DNA via the NHS to from an amide bond and the sulfhydryl group of the Cys via the maleimide group to form a stable thioehter bond. The two DNA arms are attached sequentially using HPLC purification of the intermediate products. In the first step (attachment of the first arm), we use a relatively high protein to DNA molar ratio (typically 2) in order to obtain mostly one-arm chimeras; these are separated from the smaller amount of two-identical-arms chimeras (and the uncoupled protein) on an ion exchange HPLC. The identity of the HPLC peaks is confirmed by SDS-PAGE. In the second step (attachment of the second arm), we use a relatively low one-arm-chimera to DNA molar ratio (typically 1/5) in order to obtain mostly two-arms chimeras. The final sample composition is again verified by SDS-PAGE. Finally, the two separate DNA arms are ligated to form a single 60 bp ss DNA spring end-attached to the two Cys residues on the surface of the enzyme. The procedure is described in detail in [13], [14].

The ss DNA 60mer is a very flexible polymer, but hybridization to the complementary strand rigidifies the DNA spring, which exerts a mechanical stress on the enzyme. In the experiments we compare the enzymatic activity of low stress states, realized with a nick or a ss gap in the DNA spring, and high stress states, realized with the intact ds spring. Ligated and unligated chimeras display different mobilities with SDS-PAGE; we estimate the yield of ligated constructs at >75%.

To measure the enzymatic activity of the forward reaction, we used a coupled-enzyme assay following Agarwal et al [37]. This involves three coupled reactions: GK catalyzed production of ADP and GDP from ATP and GMP, pyruvate kinase reaction where ADP and GDP react with phosphoenol pyruvate (PEP) to yield ATP, GTP and pyruvate, and lactate dehydrogenase (LDH) reaction where the pyruvate participates in the catalytic oxidization of NADH to NAD^+^. ADP and GDP production is therefore monitored by the decrease of NADH, monitored by the fluorescence at 465 nm. Levels of the coupling enzymes and their substrates were adjusted to produce linear responses not just in the fluorescence over time plots, which justifies our steady-state MM kinetics assumption, but also to ensure that increasing the amount of GK causes a corresponding increase in the speed of the reaction. A similar coupled-enzyme scheme was used to measure the rate of the backward reaction. Here ATP production of the chimera was coupled to the activity of hexokinase and glucose-6-phosphate dehydrogenase finally resulting in the reduction of 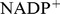, causing an observable increase in fluorescence. In the forward reaction titrations, we used 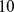 mM PEP, 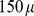
M NADH, 10 U/mL pyruvate kinase, and 13 U/mL LDH. For the reverse reaction, we used: 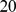 mM glucose, 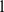 mM 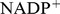, 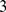 U/mL glucose-6-phosphate dehydrogenase, and 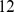 U/mL hexokinase.
